# A Multi-Disciplinary MRI Assessment May Optimize the Evaluation of Chondral Lesions in Acute Ankle Fractures: A Prospective Study

**DOI:** 10.3390/diagnostics13203220

**Published:** 2023-10-16

**Authors:** Ali Darwich, Dominik Nörenberg, Julia Adam, Svetlana Hetjens, Andreas Schilder, Udo Obertacke, Sascha Gravius, Ahmed Jawhar

**Affiliations:** 1 Department of Orthopedic and Trauma Surgery, University Medical Centre Mannheim, Medical Faculty Mannheim, University of Heidelberg, Theodor-Kutzer-Ufer 1–3, 68167 Mannheim, Germany; juliaadam@t-online.de (J.A.); andreas.schilder@medma.uni-heidelberg.de (A.S.); udo.obertacke@umm.de (U.O.); sascha.gravius@umm.de (S.G.); jawhar_ahmed@yahoo.de (A.J.); 2 Department of Radiology and Nuclear Medicine, University Medical Centre Mannheim, Medical Faculty Mannheim, University of Heidelberg, Theodor-Kutzer-Ufer 1–3, 68167 Mannheim, Germany; dominik.noerenberg@medma.uni-heidelberg.de; 3 Institute of Medical Statistics and Biomathematics, University Medical Centre Mannheim, Medical Faculty Mannheim, University of Heidelberg, Theodor-Kutzer-Ufer 1–3, 68167 Mannheim, Germany; svetlana.hetjens@medma.uni-heidelberg.de; 4 Department of Orthopedics, Traumatology and Sports Medicine, Marienhaus Hospital Hetzelstift/Teaching Hospital University Mainz, Stiftstraße 10, 67434 Neustadt an der Weinstraße, Germany

**Keywords:** ICRS, chondral lesions, ankle fracture, MRI, radiologist, orthopedic surgeon

## Abstract

Chondral lesions (CL) in the ankle following acute fractures are frequently overlooked immediately after the injury or diagnosed at a later stage, leading to persistent symptoms despite successful surgery. The literature presents a wide range of discrepancies in the reported incidence of CLs in acute ankle fractures. The objective of this prospective study is to provide a precise assessment of the occurrence of chondral lesions (CLs) in acute ankle fractures through MRI scans conducted immediately after the trauma and prior to scheduled surgery. Furthermore, the study aims to highlight the disparities in the interpretation of these MRI scans, particularly concerning the size and extent of chondral damage, between radiologists and orthopedic surgeons. Over the period of three years, all patients presenting with an unstable ankle fracture that underwent operative treatment were consecutively included in this single-center prospective study. Preoperative MRIs were obtained for all included patients within 10 days of the trauma and were evaluated by a trauma surgeon and a radiologist specialized in musculoskeletal MRI blinded to each other’s results. The location of the lesions was documented, as well as their size and ICRS classification. Correlations and kappa coefficients as well as the *p*-values were calculated. A total of 65 patients were included, with a mean age of 41 years. The evaluation of the orthopedic surgeon showed CLs in 52.3% of patients. CLs occurred mainly on the tibial articular surface (70.6%). Most talar lesions were located laterally (11.2%). The observed CLs were mainly ICRS grade 4. According to the radiologist, 69.2% of the patients presented with CLs. The most common location was the talar dome (48.9%), especially laterally. Most detected CLs were graded ICRS 3a. The correlation between the two observers was weak/fair regarding the detection and classification of CLs and moderate regarding the size of the detected CLs. To enhance the planning of surgical treatment for ankle chondral lesions (CLs), it may be beneficial to conduct an interdisciplinary preoperative assessment of the performed scans. This collaborative approach can optimize the evaluation of ankle CLs and improve overall treatment strategies.

## 1. Introduction

With a yearly incidence of 0.1 to 0.2%, ankle fractures are considered one of the most common injuries affecting the lower limbs [1,2,3]. Operative treatment of these unstable injuries with open reduction and internal fixation (ORIF) has been proven to deliver good to excellent results, since it is the most reliable way to reach anatomical alignment and stable fixation, thus restoring joint stability [2,4]. Unfortunately, several patients continue to suffer from various complaints, including recurring swelling, limited range of motion, or constant pain, even when the objectives of ORIF have been successfully achieved [5,6,7]. One of the possible hypotheses explaining these complaints are concomitant chondral lesions (CLs) that may occur during the initial trauma, since trauma has frequently been proven to be the leading cause of CLs [8]. The diagnosis of such lesions is often missed directly after the trauma or is diagnosed late, which leads to joint degeneration, chronic pain, and eventually posttraumatic osteoarthritis in 14% to 50% of patients [9,10,11,12,13]. Compared to the other joints of the lower limbs, the ankle is the joint where posttraumatic osteoarthritis most commonly occurs [13].

The reported incidence of CLs in acute ankle fractures in the literature shows a very broad range of discrepancy. A somewhat more accurate estimation of this incidence was described in a meta-analysis by Darwich et al. [14], where a mean value of 58 ± 25% (17–100) was observed, based on 19 included studies [1,9,10,11,15,16,17,18,19,20,21,22,23,24,25,26,27,28,29]. The incidence, however, varies widely according to the diagnostic method. In the same meta-analysis [14], the mean incidence was 65 ± 21% (20–100) in the 16 studies [1,10,11,15,16,18,19,20,21,22,24,25,26,27,28,29] where arthroscopy was used as diagnostic method and decreased to 19 ± 5% (17–33) in the only two available studies [9,17] where magnetic resonance imaging (MRI) was the diagnostic tool. The first MRI-based study [17] included 21 prospectively examined patients and reported an incidence of CL of 33%. The second MRI-based study [9] included 153 retrospectively analyzed patients and reported a lower incidence of 17%, despite the fact that the same diagnostic method was used. The discrepancy may be due to the fact that Boraiah et al. [9] only considered lesions of the talar dome, whereas in the study of Elsner et al. [17] all CLs, including those of the tibial and fibular articular surfaces, were included.

The association between CLs after ankle sprains and those in patients with chronic instability have been extensively investigated in the literature; however, the data describing the incidence of CLs in acute ankle fractures based on MRI are very scarce.

The assessment of preoperative MRI scans by radiologists is essential for the orthopedic surgeon in the choice of the most appropriate treatment method. Donners et al. [30] showed that orthopedic surgeons routinely consult radiology reports for most imaging studies. The confirmation of the presence of CLs and the evaluation of their size, location, and depth can lead to a radical change from a non-surgical treatment to an arthroscopic or even open surgical approach [31]. The accuracy of lesion estimation and grade of agreement between the two disciplines is therefore crucial for treatment success [32,33]. In cases of disagreement, the orthopedic surgeon should decide between the radiologist’s evaluation and his own, since literature data in this regard do not show a clear superiority of the interpretation accuracy of a specific discipline.

Thus, the aim of this prospective study is to deliver an accurate estimation of the incidence of CLs in acute ankle fractures based on MRI scans performed directly after trauma and before the planned surgery. In addition, the study seeks to show the difference in interpretation of these MRI scans between radiologists and orthopedic surgeons, especially regarding the evaluation of the size and extent of chondral damage.

## 2. Materials and Methods

### 2.1. Study Population

Over a period of three years, all patients presenting with an unstable or incongruent ankle fracture that had undergone operative treatment were consecutively included in this single-center prospective diagnostic study. Exclusion criteria included open fractures, osteoarthritis of the ankle joint, additional injuries in the same extremity or polytrauma patients, pathologic fractures due to an underlying malignancy, and rheumatoid arthritis. Also excluded were patients who did not provide written consent and patients with a intellectual disability or language disorder preventing them from fully understanding the trial features.

Operative treatment was performed according to AO principles and involved open reduction and internal fixation.

### 2.2. MRI Imaging Protocol and Evaluation

**Figure 1:**
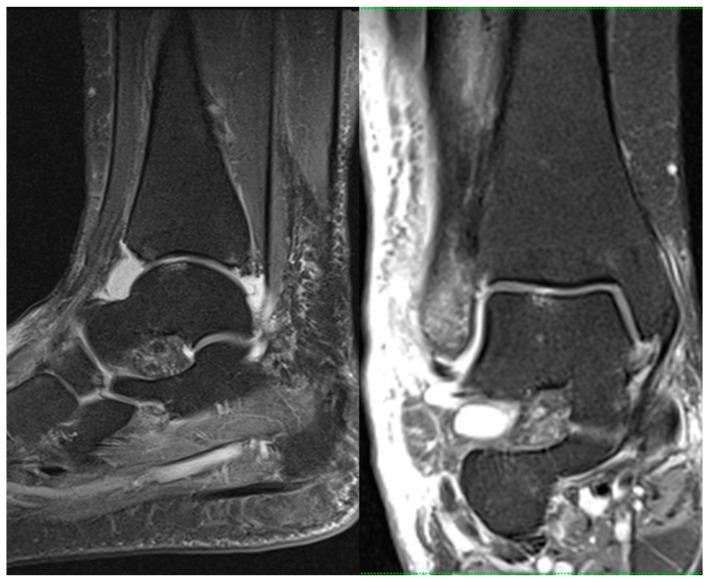
Sagittal (**left**) and coronal (**right**) proton-density-weighted turbo spin echo fat saturation MRI sequences (PD tse fs) showing CL of the talar dome in a 52-year-old female with an acute ankle fracture.

Preoperative MRIs were obtained for all included patients using a standard protocol to assess cartilage involvement including prevalence, grade, and location. All MRI scans were performed within 10 days of trauma. All scans were performed within our institution using a 1.5 T MRI scanner (Magnetom Sola, Siemens Healthineers, Germany). Standard pulse sequences included three-plane proton-density (PD) and sagittal short tau inversion recovery (STIR) sequences (Figure 1).

Patients were examined while in a supine position with the ankle in a neutral position, with use of a phased-array foot-and-ankle coil with 16 channels. We chose sagittal, axial, and coronal fat-saturated proton-density-weighted turbo spin echo (TSE) images with a TR of 3470–4000 ms, an effective TE of 40–47 ms, a slice thickness of 2 (cor, sag) or 3 mm (tra) with no interslice gap and a field of view (FOV) of 14 cm. A matrix of 512 × 384 was obtained with one or two excitations. Furthermore, a transversal T2-weighted turbo spin echo TSE image was obtained with a TR/TE of 5000/73 ms and a slice thickness of 3 mm with no interslice gap. A coronal T1-weighted TSE image was obtained with a TR of 556 ms, an effective TE of 12 ms, and a slice thickness of 2 mm.

The acquired images were evaluated by an experienced board-certified trauma surgeon with 10 years of experience and an experienced board-certified radiologist specialized in musculoskeletal MRI with 10 years of experience blinded to each other’s results and blinded to the clinical findings of the patients. Image processing software (Osirix DICOM viewer Version v.3.9.4 64-bit (Pixmeo, Geneva, Switzerland)) was used by the reviewers to measure the CLs on the performed MRI scans. Measurements included the largest lesion diameter in the coronal and sagittal planes. Depth was measured from the rim of the surrounding cartilage layer to the base of the lesion. The elliptical area formula described by Choi et al. [34] was used to calculate the lesion area.

The lesions were graded according to the International Cartilage Repair Society (ICRS) cartilage lesion classification system [35,36]:

- Grade 0: Normal
- Grade 1: Superficial lesions. Soft indentation (A) and/or superficial fissures and cracks (B)
- Grade 2: Lesions extending down to <50% of cartilage depth
- Grade 3: Cartilage defects extending down >50% of cartilage depth (A) as well as down to calcified layer (B) and down to but not through the subchondral bone (C). Blisters are included in this Grade (D)
- Grade 4: Severely abnormal. Complete cartilage lesion with perforation of the subchondral plate.

The location of the lesions was documented according to the schematic pattern proposed by Leontaritis et al. [10], dividing the talar dome into 8 zones (Zone 1 medial, zone 2 anteromedial, zone 3 anterior, zone 4 anterolateral, zone 5 lateral, zone 6 posterolateral, zone 7 posterior and zone 8 posteromedial) 1 to 8), the articular surface of the tibia in two zones (Zone T1 involving the medial malleolus and zone T2 involving the tibial plafond) and the articular surface of the fibula (Zone F1).

### 2.3. Statistical Analysis

SAS (Version 9.4 SAS Institute Inc., Cary, NC, USA) was used for all analyses. For quantitative variables, mean values and standard deviations or medians with interquartile range (IQR) were calculated. For qualitative factors, absolute and relative frequencies are presented. To compare the results, the kappa coefficient κ and the correlation coefficients (Spearman’s rs and Pearson’s r) were calculated as a measure of agreement. The kappa coefficient κ values were interpreted according to Landis et al. [37]. The correlation coefficients were interpreted according to Schober et al. [38]. Statistical significance was assumed for *p*-values less than 0.05.

### 2.4. Ethics Approval

This study was performed in line with the principles of the Declaration of Helsinki. Approval of this prospective analysis was granted by the ethics committee of clinical research at our institution (Ethikkommission II, University Medical Centre Mannheim, Medical Faculty Mannheim, Heidelberg University, Theodor-Kutzer-Ufer 1–3, 68167, Mannheim, approval no. 2016-509N-MA). Written consent was obtained from every included patient prior to their inclusion in the study.

## 3. Results

### 3.1. Baseline Patient Characteristics

A total of 65 patients were included: 37/65 males (56.9%) and 28/65 females (43.1%) with a mean age of 41.1 ± 15 years (range 15–69 years). Forty-three of the sixty-five included patients (66.2%) were smokers. The mean body mass index (BMI) of all included patients was 26.9 ± 5.1 kg/m^2^ (range 19.1–45 kg/m^2^). The mean time between trauma and MRI was 5 ± 3.8 days (range 0–10). In 33 cases (51%), the right side was involved and in 32 cases (49%), the left side.

### 3.2. Evaluation of the Orthopedic Surgeon

Of the 65 included patients, 34 (52.3%) patients showed signs of CL in the performed MRIs. A total of 45 CLs were detected: twenty-seven patients had one CL each, three patients had two CLs each, and four patients had three CLs each. Among the patients with CLs, seven of thirty-four (20.6%) involved lesions only of the talar dome, twenty-four of thirty-four (70.6%) involved lesions only of the tibial articular surface, and three of thirty-four (8.8%) involved both. The most common talar dome lesion was located laterally (11.2%). The medial, anteromedial, posteromedial, and anterior talar dome surfaces were equally involved in 4.4% of the total CLs detected and the posterolateral talar dome in 2.2% of the CLs detected. There were no lesions detected on the posterior or the anterolateral talar surfaces. The tibial plafond was the most commonly involved zone, in 53.4% of detected CLs. The articular surface of the medial malleolus was involved in 15.6% of detected CLs.

Concerning the ICRS grading, twenty-seven of all forty-five in total identified CLs (60%) were graded ICRS 4, followed by ten of forty-five (22.2%) were graded ICRS 3a, six of forty-five (13.3%) were graded ICRS 1a, and two of forty-five (4.5%) were graded ICRS 2 (Table 1). As for the size of the detected CLs, a mean lesion area of 23.1 ± 17.7 mm^2^ was measured.

### 3.3. Evaluation of the Radiologist

Of the 65 included patients, 45 (69.2%) patients showed signs of CL in the performed MRIs. A total of 70 CLs were detected: thirty patients had one CL each, eleven patients had two CLs each, and six patients had three CLs each. Among the patients with CL, 22 of 45 (48.9%) involved lesions only of the talar dome, 10 of 45 (22.2%) involved lesions only of the tibial articular surface, and 13 of 45 (28.9%) involved both. The most common talar dome CL detection was located laterally (24.2%), followed by the medial surface (12.9%) and anterior surface (10%). The remaining zones were less frequently involved (7.1% posterolateral, 2.9% anteromedial, anterolateral and posterior and 1.4% posteromedial). The tibial plafond was also the most commonly involved zone here, in 30% of detected CLs. The medial malleolus was involved in 5.7% of the detected CLs.

Concerning the ICRS grading, twenty-seven of the seventy (38.6%) total identified CLs were graded ICRS 3a, followed by twenty-one of seventy (30%) were graded ICRS 2, eighteen of seventy (25.8%) were graded ICRS 4, and four of seventy (5.6%) were graded ICRS 1b (Table 2). Regarding the size of the detected CLs, a mean lesion area of 20.8 ± 12.8 mm^2^ was measured.

The fibular articular surface did not show any significant CLs in the evaluations of both observers. Although the included fractures were formally intraarticular in nature, there were no significant CLs detected in the MRIs of the remaining patients.

### 3.4. Correlation Analysis

The correlation analysis of the gradings of the detected CLs according to the ICRS classification system between the two observers showed significant correlations regarding lesions in talar zones 3, 5, and 6 (*p* < 0.0001, *p* = 0.0091 and *p* < 0.0001, respectively) as well as in tibial zones T1 and T2 (*p* = 0.0155 and *p* = 0.0004) (Table 3). The correlation was moderate for talar zones 3 and 6 and tibial zone T2 (rs = 0.53854, rs = 0.45424 and rs = 0.42725, respectively) and weak for talar zone 5 (rs = 0.32093) and tibial zone T1 (rs = 0.29913). The interobserver agreement was similarly moderate for talar zone 3 (κ = 0.4227), fair for talar zones 5 and 6 and tibial zone T2 (κ = 0.2109, 0.3211 and 0.3030, respectively), and slight for tibial zone T1 (κ = 0.1384).

Regarding the size evaluation of the detected CLs, significant correlations between the assessments of both observers were found for talar zones 1, 3, and 6 (*p* = 0.0018, *p* = 0.0014 and *p* < 0.0001, respectively) as well as for tibial zone T2 (*p* < 0.0001). The correlation was moderate for talar zone 6 (r = 0.58661) and tibial zone T2 (r = 0.52922) and weak for talar zones 1 and 3 (r = 0.37943 and r = 0.38706, respectively (Table 3)).

## 4. Discussion

An optimal assessment of the form and extent of cartilage injury in the setting of acute ankle fractures is essential in the choice of the most appropriate treatment algorithm and consequently in the improvement of the long-term outcome [9]. Because of its superior soft tissue contrast, MRI is considered to be the best non-invasive diagnostic modality to evaluate joint cartilage [39]. In this rationale, the interpretation of MRI findings may have a substantial effect on the treatment algorithm to be chosen. Rangger et al. [40] evaluated the effect of preoperative MRI findings on surgical decisions in patients with suspected meniscal lesions and found that in 34% of cases, these findings altered the choice of surgical therapy. Therefore, the main objective of this study was to evaluate the interpretation of preoperative MRI scans of patients with acute ankle fractures and identify possible discrepancies between radiologists and orthopedic surgeons, especially regarding CL detection and assessment.

Differences in the interpretation of diagnostic examinations between radiologists and orthopedic surgeons were analyzed in several previous studies, especially involving conventional radiographs at emergency departments. In this context, discrepancies in the interpretation of conventional radiographs of up to 8–11% [41,42,43] in the adult population and 3.7% [44] in the pediatric population were observed. These discrepancies led to a change in the treatment option in 1–3% of cases [41,42,43]. In parallel, inconsistent readings and interpretations of MRI scans between orthopedic surgeons and radiologists have also been investigated. Van Grinsven et al. [31] compared the accuracy in the interpretation of magnetic resonance arthrography (MRA) imaging of experienced musculoskeletal radiologists and experienced orthopedic shoulder surgeons in patients with traumatic anterior shoulder instability and found a superior accuracy in the assessment of orthopedic surgeons in both included medical centers (79.7% vs. 75.9% and 72.7% vs. 69.8%). Halma et al. [32] investigated interdisciplinary agreement in the interpretation of MRIs of the shoulder between radiologists and orthopedic surgeons, which was found to be poor in the detection of Bankart lesions (κ −0.07 to −0.02) and fair in the detection of impingement (κ value 0.15 to 0.29). Schreinemachers et al. [45,46] evaluated the agreement of interpretations of MRAs between the same two disciplines regarding the detection of partial-thickness supraspinatus tendon tears in one study and the detection of anteroinferior labro-ligamentous lesions in a second study and observed a moderate agreement in both studies (κ value 0.48 to 0.68 and 0.44 to 0.62, respectively). Cavalli et al. [47] evaluated the interobserver agreement between two radiologists and two orthopedic surgeons in the assessment of CL of the knee and observed a fair agreement between the interpretations of the two disciplines (κ value 0.17).

In the current study, ankle CLs in acute ankle fractures were observed in 52.3% of the cases according to the orthopedic surgeon and in 69.2% of the cases according to the radiologist. These results align with previous studies that have investigated CLs in different joints. In this study, the correlation analysis for the interpretation of ankle CLs in patients with acute ankle fractures between radiologists and orthopedic surgeons varied from weak to fair when classifying CLs according to the ICRS classification. However, the correlation improved to a moderate level when evaluating the size of the identified CL.

One way to explain these discrepancies is the relatively lower accuracy of MRI in the evaluation of partial- or full-thickness cartilage lesions in general, when compared to other soft tissue injuries. As an example, data of the literature investigating the accuracy of MRI in the detection of knee pathologies show a higher accuracy for meniscal tears (90%) and cruciate ligament tears (84 to 100%) in comparison to partial-thickness cartilage lesions (80%) and full-thickness cartilage defects (63%) [48,49]. In a meta-analysis [50] including eight studies, the overall sensitivity, specificity, diagnostic odds ratio, positive likelihood ratio, and negative likelihood ratio of MRI in detecting and grading knee CLs was found to be 75%, 94%, 47, 12.5, and 0.27, respectively. Similarly, the sensitivity and specificity of MRI in the detection of CLs of the hip were shown to be 86% and 83% [51]. Joshy et al. [52] examined the accuracy of MRI in the diagnosis of CLs in the ankle joint and observed a 100% specificity and positive predictive value, a 69% negative predictive value, and an overall accuracy of 83%. However, evaluation by the orthopedic surgeon is usually based on a prior clinical diagnosis or at least a suspected clinical diagnosis resulting from an earlier knowledge of the patient´s history and the findings of a physical examination, which would obviously explain such evaluation discrepancies [31]. The positive effect of clinical information on the accuracy of MRI reports has been documented in several previous studies [53,54,55].

Another factor for the development of such discrepancies is the fact that operative treatment performed after an MRI scan provides orthopedic surgeons with some sort of continuous and direct feedback confirming or excluding the MRI findings, which enhances their focus mainly on surgery-relevant abnormalities. Radiologists, on the other hand, lack that feedback and regard the MRI scan as one whole element focusing on every irregularity or aberration [31]. This leads the orthopedic surgeons to often look for pathologies that can be treated surgically. After finding this pathology, many would stop looking for further pathologies, knowing that a more accurate assessment can be made at the time of the surgery [56].

Our study has several limitations.

First, our study shows a limited number of patients enrolled, potentially diminishing the statistical significance of certain measured correlations.

Second, there was an absence of interobserver assessment between observers from the same discipline, such as multiple radiologists or surgeons evaluating the data among themselves.

Being blinded to patients’ clinical data and age, the observers may have had misinterpreted some of the preexisting degenerative changes in the detected CL, which may be considered as an additional imitation of the study.

Nevertheless, this is the first prospective study to investigate the interdisciplinary discrepancies between radiologists and orthopedic surgeons in the interpretation of ankle MRI regarding detection and evaluation of ankle CL in the setting of acute fractures.

## 5. Conclusions

The current study demonstrates a weak to fair correlation and agreement between radiologists and orthopedic surgeons when interpreting preoperative MRI scans of patients with acute ankle fractures. This applies specifically to the detection and classification of ankle cartilage lesions (CLs) based on the ICRS classification. However, a moderate correlation and agreement were observed in evaluating the size of the identified CL. To optimize the evaluation of ankle CLs and improve the planning of surgical treatment, it is recommended to conduct an interdisciplinary preoperative assessment of the performed scans.

## Figures and Tables

**Table 1:** Distribution of CLs in each zone and their ICRS classification according to the orthopedic surgeon.

Zone	ICRS 1a	ICRS 1b	ICRS 2	ICRS 3a	ICRS 4	Total
**1**	-	-	1	-	1	**2**
**2**	1	-	-	1	-	**2**
**3**	-	-	-	2	-	**2**
**4**	-	-	-	-	-	**0**
**5**	2	-	-	3	-	**5**
**6**	-	-	-	1	-	**1**
**7**	-	-	-	-	-	**0**
**8**	1	-	1	-	-	**2**
**F1**	-	-	-	-	-	**0**
**T1**	-	-	-	1	6	**7**
**T2**	2	-	-	2	20	**24**
**Total**	**6**	**0**	**2**	**10**	**27**	**45**

**Table 2:** Distribution of CLs in each zone and their ICRS classification according to the radiologist.

Zone	ICRS 1a	ICRS 1b	ICRS 2	ICRS 3a	ICRS 4	Total
**1**	-	1	4	2	2	**9**
**2**	-	-	-	1	1	**2**
**3**	-	-	3	4	-	**7**
**4**	-	-	1	1	-	**2**
**5**	-	2	6	9	-	**17**
**6**	-	1	2	2	-	**5**
**7**	-	-	1	1	-	**2**
**8**	-	-	-	-	1	**1**
**F1**	-	-	-	-	-	**0**
**T1**	-	-	1	2	1	**4**
**T2**	-	-	3	5	13	**21**
**Total**	**0**	**4**	**21**	**27**	**18**	**70**

**Table 3:** Correlation between the evaluations of the orthopedic surgeon and the radiologist regarding the ICRS classification in each zone and the size of the CLs.

Zone	ICRS Classification			Size Evaluation	
	Kappa Value	Spearman Correlation	*p* Value	Pearson Correlation	*p* Value
**1**	0.0593	0.20187	0.1068	0.37943	*0.0018*
**2**	−0.0196	−0.03174	0.8018	−0.02830	0.8230
**3**	0.4227	0.53854	*<0.0001*	0.38706	*0.0014*
**4**	-	-	-	-	-
**5**	0.2109	0.32093	*0.0091*	0.07299	0.5634
**6**	0.3211	0.45424	*0.0001*	0.58661	*<0.0001*
**7**	-	-	-	-	-
**8**	−0.0104	−0.02227	0.8602	−0.01940	0.8781
**T1**	0.1384	0.29913	*0.0155*	0.15072	0.2307
**T2**	0.3030	0.42725	*0.0004*	0.52922	*<0.0001*
**F1**	-	-	-	-	-

## Data Availability

Data supporting the findings of this study are available from the corresponding author [A.D.] on request.
